# Engaging “No Wrong Door” Stakeholders to Improve Awareness, Access and Experiences

**DOI:** 10.1093/geroni/igaf122.3429

**Published:** 2025-12-31

**Authors:** Sara Murphy, Jennifer Severance, Douglas Mains, Esme Anaab, R Andrew Yockey

**Affiliations:** University of North Texas Health Science Center, Fort Worth, Texas, United States; University of North Texas Health Science Center - Fort Worth, TX, Fort Worth, Texas, United States; The University of North Texas Health Science Center at Fort Worth, Fort Worth, Texas, United States; The University of North Texas Health Science Center at Fort Worth, Fort Worth, Texas, United States; University of Mississippi, Oxford, Mississippi, United States

## Abstract

Many individuals with chronic comorbidities and functional limitations rely on a patchwork of long term services and supports (LTSS) to access health care, social services, and personal care as they age. However, individuals trying to navigate LTSS face a complex and often fragmented myriad of options that can delay care or lead to inadequate support or unmet needs. In 2012, the No Wrong Door (NWD) Model was implemented by states as a network of local LTSS access points designed to provide person-centered and comprehensive information, assessment, and services. In 2023, the state of Texas issued a survey to determine current awareness, perceptions, and experiences associated with the state’s NWD system. In collaboration with multiple state organizations, a survey was designed around the key elements of the NWD framework and distributed to consumers aged 50 and over, caregivers, people with disabilities, veterans and LTSS providers. Of 4,642 survey respondents, 56% identified as aged 50 and over, 22% as LTSS providers, and 20% as caregivers. Responses suggest positive experiences overall with NWD services. Consumers rated confusion (72%), long wait times (73%), not knowing how to access help (69%), and ineligibility (56%) as the most common problematic experiences with the NWD system. Most consumers (75%) and providers (66%) were unaware of the NWD System in their local communities. Recommendations are provided to guide the state in streamlining processes and engaging stakeholders to improve the consumer experience. Findings can inform policies that help states optimize awareness of and coordination between entry points.

